# The Perception of Health Care Practitioners Regarding Telemedicine During COVID-19 in Saudi Arabia: Mixed Methods Study

**DOI:** 10.2196/47065

**Published:** 2023-09-28

**Authors:** Heba Alqurashi, Rafiuddin Mohammed, Amany Shlyan AlGhanmi, Farhan Alanazi

**Affiliations:** 1 Public Health Department College of Health Sciences Saudi Electronic University Dammam Saudi Arabia; 2 Health Informatics Department College of Health Sciences Saudi Electronic University Riyadh Saudi Arabia; 3 Health Informatics Department College of Health Sciences Saudi Electronic University Jeddah Saudi Arabia

**Keywords:** telemedicine, health care practitioners, COVID-19, Saudi Arabia, mobile phone

## Abstract

**Background:**

Telemedicine is a rapidly evolving field that uses information and communication technology to provide remote health care services, such as diagnosis, treatment, consultation, patient monitoring, and medication delivery. With advancements in technology, telemedicine has become increasingly popular during the COVID-19 lockdown and has expanded beyond remote consultations via telephone or video to include comprehensive and reliable services. The integration of telemedicine platforms can enable patients and health care providers to communicate more efficiently and effectively.

**Objective:**

This study aims to investigate the awareness, knowledge, requirements, and perceptions of health care practitioners in Saudi Arabia during the pandemic health crisis from the end-user perspective. The findings of this study will inform policy makers regarding the sustainability of telemedicine and how it affects the process of provision of health care and improves the patients’ journey.

**Methods:**

This study adopted a mixed methods design with a quantitative-based cross-sectional design and qualitative interviews to assess the perceptions of various health care professionals working in outpatient departments that have a telemedicine system that was used during the COVID-19 pandemic. For both approaches, ethics approval was obtained, and informed consent forms were signed. In total, 81 completed questionnaires were used in this study. In the second phase, general interviews were conducted with managerial staff and health care professionals to obtain their view of telemedicine services in their hospitals.

**Results:**

The study revealed that most participants (67/81, 83%) were familiar with telemedicine technology, and the study proved to be statistically significant at *P*<.05 with a proportion of the participants (52/81, 64%) believing that continuous training was essential for its effective use. The study also found that consultations (55/153, 35.9%) and monitoring patients (35/153, 22.9%) were the major components of telemedicine used by health care professionals, with telephones being the most commonly used mode of interaction with patients (74/117, 63.2%). In addition, 54% (44/81) of the respondents expressed concerns about patient privacy and confidentiality, highlighting this as a major issue. Furthermore, the majority of participants (58/81, 72%) reported the necessity of implementing national standards essential for telemedicine technology in Saudi Arabia. The interviews conducted as part of the study revealed 5 major themes: culture, barriers and difficulties, communication, implementation, and evaluation. These themes highlighted the importance of a culture of acceptance and flexibility, effective communication, and ongoing evaluation of telemedicine technologies in health care systems.

**Conclusions:**

This study provides a crucial message with insights into the perceptions and experiences of health care professionals with telemedicine during the COVID-19 pandemic in Saudi Arabia.

## Introduction

### Background

Telemedicine is a remote health care practice that uses information and communication technology to provide quality medical services, including diagnosis, treatment, consultation, remote patient monitoring, and web-based drug delivery [1]. Technological developments and increasingly advanced infrastructure have made telemedicine increasingly popular throughout the world [2]. Telemedicine was initially limited to remote medical consultations via telephone or video [3]. However, with advances in technology, such as telemedicine software and remote medical devices, telemedicine has evolved into a more comprehensive and reliable service [4]. Telemedicine can be used as an effective data management system to efficiently store and process patient medical data. This makes it easier for health care service providers to provide better care [5].

An integrated telemedicine platform can make it easy and quick for patients and health care providers to communicate with each other [6]. This includes mobile apps, web portals, and appointment management systems. With the development of technology and the increasingly widespread use of telemedicine, telemedicine services are expected to become more effective and efficient [7]. However, the challenges and drawbacks of telemedicine must also be considered and addressed to ensure effective and high-quality health services [8].

The entire world is currently facing a new disease, the novel COVID-19, which started its journey in China in 2019 [9] and spread all over the world in an explosive pandemic [10]. This has led many countries and economies to adapt to many technical tools that were crucial for controlling, monitoring, and managing the contagion [3]. The Kingdom of Saudi Arabia spared no efforts in implementing infection containment measures while also meeting the health care needs of its society [11,12].

Before the pandemic, the Ministry of Health launched its eHealth strategies and initiatives in 2011 [13]. Since then, the Ministry of Health has been working to deploy telemedicine services, and because of these efforts, various platforms are currently providing telemedicine services. The best-known platforms for providing patients direct access to telemedicine services are the Seha App and 937-telephone health services. These 2 platforms cover consultations, appointment scheduling, web-based clinics (through the Seha App), and more. When compared with the period during the pandemic, the use of both platforms was significantly lower before the outbreak [14,15]. Telemedicine is an emerging technology that shows undeniable value in satisfying consumers’ health needs at any time and place through cost-effective models [16]. A variety of telecommunication tools were used, including web-based clinics, teleconsultations, e-prescriptions and medication refills through home delivery, mobile apps, and distance monitoring, with a final option for admission into special units if required [15].

Telemedicine is currently gaining popularity because of the COVID-19 pandemic [17]. Many patients are unable or unwilling to visit hospitals or clinics because of the risk of spreading the virus. Therefore, telemedicine is a very useful solution for maintaining continuity of medical care during the pandemic [18]. Despite the high use of these services, evidence about the effectiveness of telemedicine care and its delivery model, in addition to the percentage of failed projects, eventually have reached 75% and approximately 95% in middle income countries because of end-user adoption barriers [19]. Despite the literature indicating a high level of knowledge and awareness among health care providers regarding telecommunication alternatives, the continued challenges in implementing telemedicine can be attributed to several possible reasons [20,21].

### Objectives

Owing to the aforementioned reasons, this study aims to investigate the awareness, knowledge, requirements, and perceptions from the end-user perspectives of health care practitioners in Saudi Arabia during the pandemic health crisis.

This aim will be achieved by completing the following objectives: (1) understanding the awareness of health care workers regarding telemedicine, (2) investigating the effect of telemedicine on health care during COVID-19, and (3) exploring the perception of health care workers and managers regarding the use of telemedicine and its use during COVID-19.

To the best of our knowledge, this is the first study in Saudi Arabia that focuses mainly on the effectiveness and efficiency of telemedicine technology from the perspective of health practitioners during the COVID-19 outbreak.

## Methods

### Design and Setting

This study adopted a mixed methods design with a quantitative-based cross-sectional design and qualitative interviews to assess the perception of various health care professionals working in outpatient departments that have a telemedicine system that was used during the COVID-19 pandemic period, which includes during the lockdown period and afterward. The survey was conducted at 2 major hospitals based in the central and eastern regions of Saudi Arabia. The study was conducted in 2 phases. In the first phase, the various health care professionals included in this study were interns, residents, technicians, various health specialists, and consultants working in different outpatient departments. These professionals were selected using a random convenience sampling procedure. This study was conducted from October 2021 to April 2022. Professionals with <1 year of experience in telemedicine and those aged <18 years were excluded. Professionals of both nationalities (Saudis) and nonnationalities (non-Saudis) were included in this study. A web-based survey tool was sent as an invitation link to participate in the study via their respective department’s email. In total, 81 completed questionnaires were used in this study. In the second phase, one-to-one interviews were conducted with telemedicine and outpatient department managers to provide a managerial and professional view of telemedicine services in their hospitals.

### Questionnaire Design

For the first phase of the study, a questionnaire was adopted from previously published research articles and modified to align with this study’s objectives (see Multimedia Appendix 1 for the survey) [4,6]. A total of 6 expert members who had experience in the field of telemedicine evaluated the questionnaire for data quality, and it was adopted in English. The questionnaire was composed of seven sections: (1) demographic characteristics of the participants, (2) knowledge of telemedicine, (3) perception of the advantages of telemedicine technology, (4) perception of the disadvantages of telemedicine technology, (5) perception of the necessity of telemedicine technology, (6) perception of issues affecting telemedicine, and (7) perceptions of telemedicine effectiveness obtained from patient feedback. The perceptions of each domain were rated using a 3-point Likert scale (agree, neutral, and disagree) [22]. For the second phase of the study, interviews with open-ended questions were conducted using previously published research questions with modifications [8]. It was used to assess the managerial views of the heads of departments with respect to telemedicine challenges during the COVID-19 pandemic crisis in their hospitals. Approximately 5 different heads of departments in both hospitals were interviewed to develop a conceptual theme framework. Before the study began, a pilot study was conducted to measure the validity and internal consistency of the questionnaire.

### Ethical Considerations

The study received research project approval from the Deanship of Scientific Research at Saudi Electronic University (7870-HS-2021) and ethical clearance from both hospitals’ institutional review boards (21-393E and EXT0391). The participants were required to sign an informed consent form before completing the questionnaire and undergoing the interviews, which provided a brief description of the study, including significance, and assurance of full confidentiality of their data and their freedom to leave or not answer if they wish to do so.

### Data Analysis

SPSS (version 24; IBM Corp) was used for data analysis [23]. Descriptive statistics were used to generate summary tables for various study variables [24]. All parameters were expressed as frequencies and percentages. Differences were considered statistically significant at *P*<.05 [25]. Moreover, the interviews were transcribed and analyzed using the NVivo (release 1.0; QSR International) computer software package to develop themes from the views of the heads of departments [26].

## Results

### Overview

A total of 81 health professionals, of which 38 (47%) were men and 43 (53%) were women, were included in the analysis. Most of the participants were Saudis (54/81, 67%). The remaining non-Saudi participants were Indians (10/81, 12%), Sudanese (7/81, 6%), Egyptian (5/81, 6%), Americans (2/81, 2%), Philippines (1/81, 1%), Jordan (1/81, 1%), and Yemen (1/81, 1%). Half of the participants (42/81, 52%) were aged between 18 and 30 years. The majority of the health professionals were residents (25/81, 31%), followed by specialists (21/81, 26%), and the least were consultants (7/81, 9%). With regard to experience, >5years of experience in the field of telemedicine was reported by 27% (22/81) of the participants, and the highest number of patients seen through telemedicine channels per day was between 20 and 40 patients (47/81, 58%). Furthermore, the duration of consultation per day ranged between 1 hour to 3 hours (37/81, 46%) and 3 hours to 5 hours (29/81, 36%). Table 1 presents the demographic characteristics of the participants.

**Table 1:** Demographic characteristic of the participants (N=81).

Characteristics		Participants, n (%)
**Gender**		
	Men	38 (47)
	Women	43 (53)
**Age (years)**		
	18-30	42 (52)
	31-40	24 (30)
	41-50	11 (14)
	51-60	4 (5)
	≥60	0 (0)
**Nationality**		
	Saudi	54 (67)
	Non-Saudi	27 (33)
**Professional level**		
	Intern	17 (21)
	Resident	25 (31)
	Technician	11 (14)
	Specialist	21 (26)
	Consultant	7 (9)
**Experience (years)**		
	2	30 (37)
	3	19 (24)
	4	10 (12)
	>5	22 (27)
**Number of patients seen through the telemedicine channel per day**		
	20-40	47 (58)
	41-60	27 (33)
	61-80	4 (5)
	81-100	1 (1)
	>100	2 (3)
**Consultation duration per day**		
	1-3 hours	37 (46)
	4-5 hours	29 (36)
	6-8 hours	9 (11)
	>8 hours	6 (7)

### Knowledge, Advantages, and Disadvantages of Telemedicine During COVID-19

In total, 83% (67/81) of the health professionals reported that they were familiar with telemedicine technology, of which consultants (7/81, 100%) and technicians (10/81, 91%) showed the highest level of knowledge. Approximately 27% (22/81) of the participants expressed the view that they were neutral with regard to telemedicine guidelines, and the majority of participants (52/81, 64%) agreed that there must be continuous training as an essential requirement in the use of telemedicine. The impact of telemedicine use did not show a significant difference between the different health professions (Table 2). The results showed that 75% (61/81) of the health professionals are aware of the benefits of telemedicine. Regarding the influence of telemedicine on users’ satisfaction, the majority agreed on its influence (51/81, 63%) and at the same time found it to be neutral (21/81, 26%; *P*=.049). Moreover, the study revealed that telemedicine can save clinicians’ time (58/81, 72%) and provide faster and better medical care (54/81, 67%; Table 3). In contrast, regarding the disadvantages of telemedicine, the findings conveyed were neutral about doctor-patient relationship disruption (29/81, 36%), and 44% (36/81) of the participants disagreed on that. The use of telemedicine technology reduced the effectiveness of patient care with a slight significance (*P*=.03). Finally, half of the respondents disagreed that telemedicine provided unauthorized access to patient medical information (40/81, 49%), increased malpractice in health care (39/81, 48%), and increased hospital expenses (39/81, 48%; Table 4).

**Table 2:** Health care professionals’ knowledge of telemedicine (N=81).

Questions and level		Intern, n (%)			Resident, n (%)		Technician, n (%)		Specialist, n (%)		Consultant, n (%)		Total, n (%)		*P* value	
**I am familiar with telemedicine technology**																.93
	Agree		14 (82)	20 (80)		10 (91)		16 (76)		7 (100)		67 (83)				
	Neutral		3 (18)	5 (20)		1 (9)		4 (19)		0 (0)		13 (16)				
	Disagree		0 (0)	0 (0)		0 (0)		1 (5)		0 (0)		1 (1)				
**I am familiar with telemedicine guidelines**																.47
	Agree		12 (71)	17 (68)		10 (91)		11 (52)		3 (43)		53 (65)				
	Neutral		3 (18)	6 (24)		1 (9)		8 (38)		4 (57)		22 (27)				
	Disagree		2 (12)	2 (8)		0 (0)		2 (10)		0 (0)		6 (7)				
	Agree		9 (53)	11 (44)		10 (91)		15 (71)		7 (100)		52 (64)				
	Neutral		5 (29)	11 (44)		1 (9)		5 (24)		0 (0)		22 (27)				
	Disagree		3 (18)	3 (12)		0 (0)		1 (5)		0 (0)		7 (9)				

**Table 3:** Health care professionals’ perception of advantages of using telemedicine technology during COVID-19 (N=81).

Questions and level		Intern, n (%)	Resident, n (%)	Technician, n (%)	Specialist, n (%)	Consultant, n (%)	Total, n (%)	*P* value	
**I am familiar with the benefits of telemedicine**									.39
	Agree	12 (71)	16 (64)	11 (100)	16 (76.1)	6 (86)	61 (75)		
	Neutral	3 (18)	5 (20)	0 (0)	5 (23.8)	1 (14)	14 (17)		
	Disagree	2 (12)	4 (16)	0 (0)	0 (0)	0 (0)	6 (7)		
**Telemedicine is effective in reducing the costs of patient care in hospitals**									.29
	Agree	10 (59)	12 (48)	9 (82)	15 (71)	7 (100)	53 (65)		
	Neutral	6 (35)	6 (24)	1 (9)	4 (19)	0 (0)	17 (21)		
	Disagree	1 (6)	7 (28)	1 (9)	2 (10)	0 (0)	11 (14)		
**Telemedicine influences users’ satisfaction**									.05
	Agree	10 (59)	11 (44)	10 (91)	13 (62)	7 (100)	51 (63)		
	Neutral	3 (18)	11 (44)	1 (9)	6 (29)	0 (0)	21 (26)		
	Disagree	4 (24)	3 (12)	0 (0)	2 (95)	0 (0)	9 (11)		
**Telemedicine technology saves clinicians’ time**									.56
	Agree	12 (71)	16 (64)	11 (100)	13 (62)	6 (86)	58 (72)		
	Neutral	5 (29)	8 (32)	0 (0)	7 (33)	1 (14)	21 (26)		
	Disagree	0 (0)	1 (4)	0 (0)	1 (5)	0 (0)	2 (2)		
**Telemedicine provides faster and better medical care**									.60
	Agree	10 (59)	15 (60)	11 (100)	14 (67)	4 (57)	54 (67)		
	Neutral	5 (29)	8 (32)	0 (0)	5 (24)	3 (43)	21 (26)		
	Disagree	2 (12)	2 (8)	0 (0)	2 (10)	0 (0)	6 (7)		
**Telemedicine is an effective technology for improving patient care**									.63
	Agree	7 (41)	15 (60)	9 (82)	12 (57)	7 (100)	50 (62)		
	Neutral	6 (35)	4 (16)	1 (9)	6 (29)	0 (0)	17 (21)		
	Disagree	4 (24)	6 (24)	1 (9)	3 (14)	0 (0)	14 (17)		

**Table 4:** Health care professionals’ perception of disadvantages of using telemedicine technology during COVID-19 (N=81).

Questions and level		Intern, n (%)	Resident, n (%)	Technician, n (%)	Specialist, n (%)	Consultant, n (%)	Total, n (%)	*P* value	
**Telemedicine technology disrupts the doctor-patient relationship**									.79
	Agree	7 (41)	10 (40)	4 (36)	9 (43)	1 (14)	31 (38)		
	Neutral	7 (41)	8 (32)	4 (36)	8 (38)	2 (29)	29 (36)		
	Disagree	3 (18)	7 (28)	3 (27)	4 (19)	4 (57)	21 (26)		
**Telemedicine technology reduces the effectiveness of patient care**									.28
	Agree	3 (18)	3 (12)	6 (55)	4 (19)	1 (14)	17 (21)		
	Neutral	6 (35)	11 (44)	1 (9)	8 (38)	2 (29)	28 (35)		
	Disagree	8 (47)	11 (44)	4 (36)	9 (43)	4 (57)	36 (44)		
**Telemedicine technology causes psychological harm to the patients**									.49
	Agree	2 (12)	3 (12)	3 (27)	5 (24)	1 (14)	14 (17)		
	Neutral	5 (29)	10 (40)	3 (27)	7 (33)	0 (0)	25 (31)		
	Disagree	9 (53)	12 (48)	5 (45)	9 (43)	6 (86)	42 (52)		
**Telemedicine technology results in unauthorized access to patient medical information**									.79
	Agree	3 (18)	5 (20)	4 (36)	7 (33)	1 (14)	20 (25)		
	Neutral	3 (18)	7 (28)	3 (27)	7 (33)	1 (14)	21 (26)		
	Disagree	11 (65)	13 (52)	4 (36)	7 (33)	5 (71)	40 (49)		
**Telemedicine technology increases hospital expenses**									.20
	Agree	2 (12)	3 (12)	2 (18)	5 (25)	1 (14)	13 (16)		
	Neutral	7 (41)	3 (12)	3 (27)	9 (43)	1 (14)	23 (28)		
	Disagree	6 (35)	16 (64)	6 (55)	6 (30)	5 (71)	39 (48)		
**Telemedicine technology increases malpractice in health care**									.35
	Agree	1 (6)	5 (20)	2 (18)	9 (43)	2 (29)	19 (24)		
	Neutral	7 (41)	6 (24)	5 (46)	5 (24)	0 (0)	23 (28)		
	Disagree	9 (53)	14 (56)	4 (36)	7 (33)	5 (71)	39 (48)		

### Necessity of Using Telemedicine Technology During COVID-19

This study showed a high level of perception (54/81, 67%) and at the same time neutrality (22/81, 27%) concerning the necessity of patient care through telemedicine during COVID-19 (*P*=.13). Many health professionals were neutral about providing health in remote areas (18/81, 22%) and providing doctors with instant access to patient information (18/81, 22%). One of the important outcomes disclosed in this study was that (58/81, 72%) believed that it was necessary to implement national standards essential for telemedicine technology in Saudi Arabia (Table 5).

**Table 5:** Health care professionals’ perception of necessity of using telemedicine technology during COVID-19 (N=81).

Questions and level		Intern, n (%)	Resident, n (%)	Technician, n (%)	Specialist, n (%)	Consultant, n (%)	Total, n (%)	*P* value	
**Telemedicine technology is necessary for patient care**									.13
	Agree	10 (59)	16 (64)	9 (82)	13 (62)	6 (86)	54 (67)		
	Neutral	3 (18)	9 (36)	2 (18)	7 (33)	1 (14)	22 (27)		
	Disagree	4 (24)	0 (0)	0 (0)	1 (5)	0 (0)	5 (6)		
**Telemedicine provides health care to patients in a timely manner**									.39
	Agree	8 (47)	16 (64)	9 (82)	13 (62)	6 (86)	52 (64)		
	Neutral	6 (35)	7 (28)	2 (18)	5 (24)	1 (14)	21 (26)		
	Disagree	3 (18)	2 (8)	0 (0)	3 (14)	0 (0)	8 (10)		
**Telemedicine is essential to provide health care to underprivileged and remote areas**									.61
	Agree	9 (53)	15 (60)	8 (73)	14 (67)	6 (86)	52 (64)		
	Neutral	4 (24)	7 (28)	2 (19)	4 (19)	1 (14)	18 (22)		
	Disagree	4 (24)	3 (12)	1 (9)	3 (14)	0 (0)	11 (14)		
**Telemedicine technology provides doctors with instant access to patient information**									.22
	Agree	7 (41)	15 (60)	11 (100)	14 (67)	5 (71)	52 (64)		
	Neutral	7 (41)	5 (20)	0 (0)	4 (19)	2 (29)	18 (22)		
	Disagree	3 (18)	5 (20)	0 (0)	3 (14)	0 (0)	11 (14)		
**National standards are essential for telemedicine technology implementation in Saudi Arabia**									.62
	Agree	10 (59)	17 (68)	11 (100)	14 (67)	6 (86)	58 (72)		
	Neutral	3 (18)	6 (24)	0 (0)	4 (19)	1 (14)	14 (17)		
	Disagree	4 (24)	2 (8)	0 (0)	3 (14)	0 (0)	9 (11)		

### Issues Affecting Telemedicine Technology During COVID-19

The results revealed that 54% (44/81) of the respondents considered patient privacy and confidentiality to be a significant concern in the context of telemedicine during COVID-19. However, 46% (37/81) of the participants disagreed with the high cost of equipment, whereas 52% (42/81) disagreed with the negative attitude of staff being an issue. Interestingly, approximately an equal number of responses considered whether the lack of user-friendly software and lack of suitable training were issues among professionals (Table 6).

**Table 6:** Health care professionals’ perception of issues affecting telemedicine technology during COVID-19 (N=81).

Questions and level		Intern, n (%)	Resident, n (%)	Technician, n (%)	Specialist, n (%)	Consultant, n (%)	Total, n (%)	*P* value	
**Concerns about patient privacy and confidentiality**									.12
	Agree	8 (47)	15 (60)	8 (73)	12 (57)	1 (14)	44 (54)		
	Neutral	5 (29)	9 (36)	0 (0)	6 (29)	2 (29)	22 (27)		
	Disagree	4 (24)	1 (4)	3 (27)	3 (14)	4 (57)	15 (18)		
**High cost of equipment**									.80
	Agree	4 (24)	11 (44)	6 (55)	7 (33)	1 (14)	29 (36)		
	Neutral	2 (12)	3 (12)	2 (18)	6 (29)	2 (29)	15 (19)		
	Disagree	11 (65)	11 (44)	3 (27)	8 (38)	4 (57)	37 (46)		
**Negative attitudes of staff involved**									.39
	Agree	6 (35)	6 (24)	2 (18)	6 (29)	0 (0)	20 (25)		
	Neutral	2 (12)	6 (24)	2 (18)	8 (38)	1 (14)	19 (24)		
	Disagree	9 (53)	13 (52)	7 (64)	7 (33)	6 (86)	42 (52)		
**Lack of user-friendly software**									.33
	Agree	6 (35)	9 (36)	4 (36)	5 (24)	3 (43)	27 (33)		
	Neutral	3 (18)	6 (24)	2 (18)	10 (48)	2 (29)	23 (28)		
	Disagree	8 (47)	10 (40)	5 (45)	6 (29)	2 (29)	31 (38)		
**Lack of suitable training in the use of equipment**									.37
	Agree	3 (18)	9 (36)	4 (36)	10 (48)	2 (29)	28 (35)		
	Neutral	9 (53)	7 (28)	2 (18)	6 (29)	2 (29)	26 (32)		
	Disagree	5 (29)	9 (36)	5 (46)	5 (24)	3 (43)	27 (33)		
**Perceived increase in workload**									.47
	Agree	4 (24)	9 (36)	3 (27)	7 (33)	2 (29)	25 (31)		
	Neutral	11 (65)	10 (40)	5 (46)	8 (38)	2 (29)	36 (44)		
	Disagree	2 (12)	6 (24)	3 (27)	6 (29)	3 (43)	20 (25)		
**Lack of perceived clinical usefulness**									.70
	Agree	6 (35)	8 (32)	3 (27)	7 (33)	2 (29)	26 (32)		
	Neutral	9 (53)	10 (40)	4 (36)	8 (38)	1 (14)	32 (40)		
	Disagree	2 (12)	7 (28)	4 (36)	6 (29)	4 (57)	23 (28)		

### Effectiveness of Telemedicine Used by Patients During COVID-19

The study findings received mixed responses between agreement (39/81, 48%) and neutral (33/81, 41%) in the use of telemedicine tools by patients during consultations. Interestingly, half of the respondents (42/81, 52%) understood the medical problems of patients and provided management plans to patients (41/81, 51%). Curiously, many participants reported neutral (36/81, 44%) promptness to turn up for recall check-ups for the next visit (Table 7).

**Table 7:** Health care professionals’ perception of the effectiveness of telemedicine used by patients during COVID-19 (N=81).

Questions and level		Intern, n (%)	Resident, n (%)	Technician, n (%)	Specialist, n (%)	Consultant, n (%)	Total, n (%)	*P* value	
**Patients easily use telemedicine tools during consultations**									.79
	Agree	8 (47)	13 (52)	5 (46)	11 (52)	2 (29)	39 (48)		
	Neutral	7 (41)	10 (40)	5 (46)	7 (33)	4 (57)	33 (41)		
	Disagree	2 (12)	2 (8)	1 (9)	3 (14)	1 (14)	9 (11)		
**Clarifying medical problems to patients during consultation**									.85
	Agree	8 (47)	12 (48)	6 (55)	12 (57)	4 (57)	42 (52)		
	Neutral	7 (41)	8 (32)	5 (46)	5 (24)	3 (43)	28 (35)		
	Disagree	2 (12)	5 (20)	0 (0)	4 (19)	0 (0)	11 (14)		
**Provides appropriate management plans to patients**									.39
	Agree	5 (29)	11 (44)	7 (64)	14 (67)	4 (57)	41 (51)		
	Neutral	8 (47)	9 (36)	4 (36)	6 (29)	3 (43)	30 (37)		
	Disagree	4 (24)	5 (20)	0 (0)	1 (5)	0 (0)	10 (12)		
**Patients promptly turn up for recall checkup**									.31
	Agree	5 (29)	11 (44)	5 (46)	9 (43)	4 (57)	34 (42)		
	Neutral	6 (35)	10 (40)	6 (55)	11 (52)	3 (43)	36 (44)		
	Disagree	6 (35)	4 (16)	0 (0)	1 (5)	0 (0)	11 (14)		

### Components of Telemedicine Used and the Mode of Telemedicine Interaction

In this study, consultations (55/153, 35.9%), followed by monitoring patients (35/153, 22.9%), were the major components used by health professionals during telemedicine services, and the least used was diagnosis (14/153, 9.2%; Table 8). However, with reference to the mode of telemedicine interaction with patients during COVID-19, telephone (74/117, 63.2%) was reported to be the most used, and email (1/117, 0.9%) was the least used (Table 9). Telephone (74/117, 63.2%) was reported be the most used, followed by web-based chatting (17/117, 14.5%) and videoconferencing (11/117, 9.4%), and the least used was email (1/117, 0.9%; Table 9).

**Table 8:** Components of telemedicine used (n=153).

Characteristics	Values^a^, n (%)
Consultations	55 (35.9)
Diagnosis	14 (9.2)
Treatment	30 (19.6)
Monitoring patients	35 (22.9)
Nursing	19 (12.4)

^a^More than one choice could be selected.

**Table 9:** Mode of telemedicine interaction (n=117).

Characteristics	Values^a^, n (%)
Telephone	74 (63.2)
Video conferencing	11 (9.4)
Online chatting	17 (14.5)
Ministry of Health applications	14 (11.9)
Email	1 (0.9)

^a^More than one choice could be selected.

### Theme Framework of Interviewers

Five interviews were conducted with semistructured questions regarding COVID-19’s effect on patients, health care workers, and managers, including the use of telemedicine, effect on finances, and quality of care; the themes are listed in Textbox 1.

**Framework for interview themes.**

- Users’ acceptance among clinical staff and their willingness to use telemedicine during COVID-19
- Patients’ acceptance and their willingness to be treated by telemedicine during COVID-19
- The availability of adequate experts during the COVID-19 pandemic
- The presence of approved strategy and plans for implementing telemedicine according to the Ministry of Health guidelines during COVID-19
- Availability of the required information and communication technology infrastructure
- Availability of the required information about patients from their health records
- System quality (eg, reliability, supportability, security, interoperability, privacy, and functionality)
- Information quality (ie, its accuracy, completeness, usefulness, and relevancy)
- Ensuring the cost-effectiveness of telemedicine
- The availability of reimbursement for telemedicine services for insurance purposes
- The availability of adequate sustainable funding or financial support from government and the Ministry of Health to implement, operate, and maintain telemedicine
- Economic constraints and recession affected the telemedicine process
- Any other challenges that are barriers during COVID-19 with regard to telemedicine

**Figure 1:**
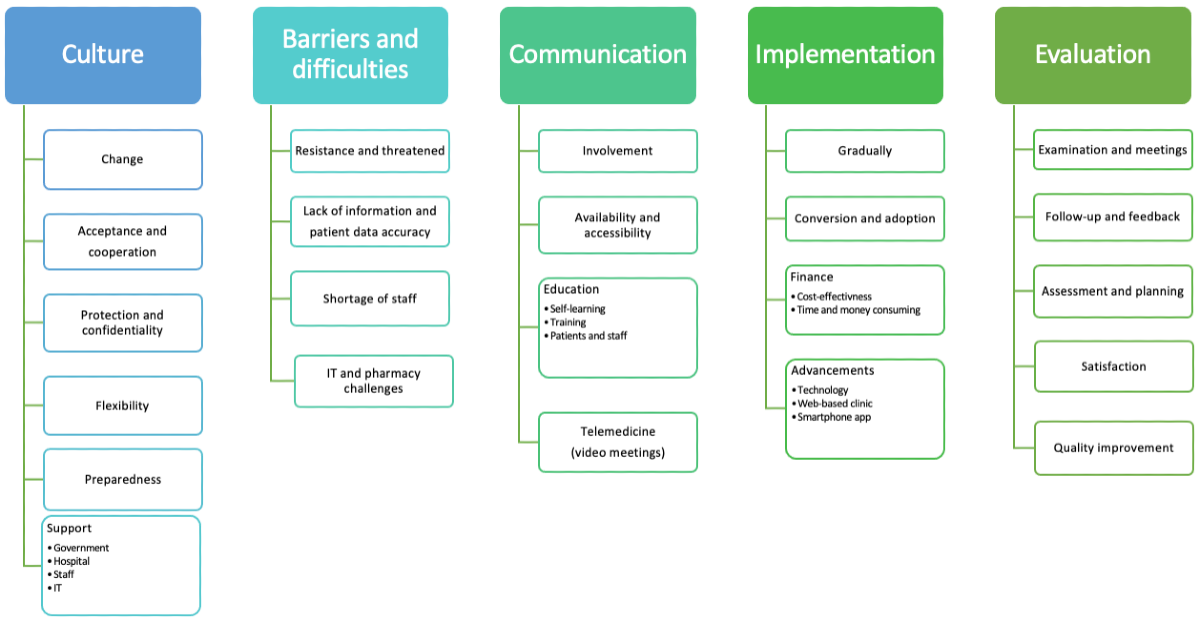
Thematic analysis of the conceptual theme framework of interviewers.

After transcription, the interviews were thematically analyzed, which resulted in 5 major themes, as shown in Figure 1.

The COVID-19 pandemic has brought about many changes in hospitals, and the culture of hospitals must adapt accordingly:

COVID-19 at the beginning was a change of culture, change of management and the delivery of patient care.

Interviewee 1

The first theme that emerged from the interviews was culture, with change being a subtheme. Patients’ acceptance of telemedicine was a smooth success, which could be attributed to the pandemic, with protection and confidentiality as high priorities and the need for flexibility as an integral part of the culture. Support was also required to sustain the system, whether external from the government or internal from the hospital administration, staff, and IT.

The second theme was barriers and difficulties. Resistance was a significant barrier; however, during COVID-19, it was also connected to people not wanting to spread the disease:

Lack of information about the disease was a major barrier but also shortage of staff which also lead to resistance to adopting new technologies.

Interviewee 2

Lack of information about the disease and the importance of patient accuracy were heightened to continue providing satisfactory care. Furthermore, the shortage of staff worldwide, coupled with pandemic precautions, was another significant barrier. IT and pharmaceutical difficulties also arose because of the lockdown and sudden changes in the country.

The third theme was communication, emphasizing the importance of involving everyone in overcoming the pandemic. Providing accessibility, education, and making telemedicine available to both staff and patients were essential aspects of this effort:

We started using telemedicine as a project prior to COVID-19, however we increased our education and awareness sessions with the patients and members during that period and had follow up meetings to discuss the accessibility of the system.

Interviewee 5

The internet allowed self-learning, and a system was in place that allowed health care providers to access patients, call them, and meet with them through video meetings to provide continuity of care during these difficult times.

The fourth theme was implementation, where the shift to telemedicine occurred gradually in most hospitals, the adoption process was integral, technological advancements were used, and finances were made available for the implementation process to occur:

The shift to telemedicine was done gradually, with weekly meetings with the staff to get feedback on any issues and meetings with IT to overcome these issues.

Interviewee 3

The fifth and final theme was evaluation, as the assessment, examinations, follow-ups, and feedback of patients and health care workers were discussed regularly, and weekly meetings were held to obtain feedback and discuss barriers, challenges, and facilities of team members to keep the system updated. Telemedicine helped with quality improvement by shifting face-to-face appointments to telemedicine, which most patients now prefer based on their evaluation and feedback of the system.

## Discussion

### Principal Findings

The primary focus of our research was to comprehensively examine the awareness, knowledge, requirements, and perceptions of health care practitioners in Saudi Arabia regarding telemedicine during the unprecedented COVID-19 pandemic. This exploration, pioneering in its focus and context, aimed to capture the effectiveness and efficiency of telemedicine technology as perceived by these professionals during the health crisis.

Our findings highlight a significant awareness of telemedicine technology among the participants. Familiarity with various telemedicine modalities suggests that health care practitioners in Saudi Arabia have been exposed to, if not actively engaged with, telemedical tools before or during the pandemic. This widespread awareness is further corroborated by other research [19,27]. A prominent result of our study was the acknowledgment of the benefits of telemedicine, such as improved time efficiency for clinicians and an enhancement in the speed and quality of medical care (Table 3). These benefits are consistent with those of previous reports [17,20]. However, concerns about patient privacy and confidentiality were prevalent, with over half of the participants (44/81, 54%) expressing these concerns (Table 6). Given Saudi Arabia’s distinct cultural context, privacy concerns are particularly accentuated owing to cultural nuances [12,28]. The findings of this study are crucial for policy makers in Saudi Arabia, emphasizing the need to address these considerations for a more sustainable future implementation of telemedicine.

Delving deeper into the details, there was a strong inclination among participants toward the establishment of national telemedicine standards in Saudi Arabia (Table 5). The study’s findings suggest a pronounced preference for consultations and patient monitoring in telemedicine modalities. Notably, telephones were identified as the primary mode of patient interaction (Tables 8 and 9). This trend aligns with global shifts, notably during the COVID-19 pandemic when telephone health services saw a marked increase in use [15]. Simultaneously, there was a noticeable increase in the use of web-based chatting (17/117, 14.5%) and videoconferencing (11/117, 9.4%), establishing mobile phones as pivotal tools for telehealth during this period.

Data from interviews highlighted various obstacles to the full adoption of telemedicine. Achieving success relies on fostering a culture of acceptance, ensuring system flexibility, maintaining effective communication, and continuously evaluating telemedicine in health care environments (Figure 1). Collectively, these results suggest that telemedicine is a cost-effective and quality-enhancing mechanism that is crucial for ensuring the continuity of medical care, particularly during crises such as the COVID-19 pandemic [6,11,19]. Nevertheless, because it is a comprehensive integration, it is essential to address current barriers, promote a receptive culture, uphold transparent communication, and constantly appraise this technological advancement [29,30].

When juxtaposing the telemedicine framework in Saudi Arabia with global benchmarks, several influential factors emerge, including health care infrastructure, technological accessibility, regulatory standards, and the adoption rate among end users, both the general public and health care professionals [31,32]. Developed countries, such as the United States, have long integrated telemedicine into their health care systems [33], offering various modalities such as videoconferencing and remote monitoring. In contrast, developing countries may face challenges such as infrastructural limitations or restricted technological access, which act as barriers to telemedicine adoption [34].

Although Saudi Arabia has established regulations for telemedicine [35], it’s journey in telehealth is still in its infancy. The nation’s cultural and religious contexts significantly influence the adoption and modality preferences of telemedicine [36]. The COVID-19 pandemic, however, served as a catalyst, significantly boosting the adoption of telemedicine. This is supported by governmental policies [27]. The future of telehealth in Saudi Arabia seems bright, with expectations of more integrated and expansive use, benefiting both the general public and health care professionals.

This study offers a nuanced understanding of the perceptions, requirements, and challenges faced by health care practitioners in Saudi Arabia regarding telemedicine during the COVID-19 pandemic. It presents a pioneering effort to highlight areas of strength and pinpoint avenues for further development and integration of telemedicine into the Saudi Arabian health care landscape.

### Implications

The study’s findings have several managerial implications for health care organizations. First, it is important for managers to provide continuous training to their staff to improve their telemedicine technology knowledge and skills. Second, managers should prioritize patient privacy and confidentiality by implementing measures to protect patients’ personal and medical information. Third, managers should ensure that telemedicine technology used in organizations is user-friendly. Fourth, health care organizations should develop national standards for telemedicine technology in their countries to ensure consistency and quality of care. Finally, managers should regularly evaluate the use of telemedicine to identify areas for improvement and to ensure that the technology meets the needs of patients and health care providers. By implementing these managerial implications, health care organizations can improve the efficiency and effectiveness of their telemedicine services, which would ultimately lead to improved patient outcomes and satisfaction.

### Limitation and Future Research

This study was conducted in only 2 hospitals in Saudi Arabia, which may not be representative of all the health care facilities in the country. The study included only health care professionals who had experience with telemedicine, which may not be representative of all health care professionals. This study relied on self-reported data, which may be subject to bias and may not accurately reflect the actual perceptions and experiences of health care professionals.

The use of random convenience sampling may introduce bias and limit the generalizability of the results. In addition, the use of a web-based survey tool may introduce a response bias, as those who are more comfortable with technology may be more likely to participate. Moreover, the diversity of perspectives included in this study was not accounted for.

Future research should investigate the perceptions and experiences of health care professionals with telemedicine in other health care facilities in Saudi Arabia to determine whether the findings are generalizable. Future research could also include health care professionals who have little or no experience with telemedicine to determine the barriers and challenges that need to be addressed to increase adoption.

It would be beneficial to conduct a study that includes patients’ perceptions and experiences with telemedicine to gain a more comprehensive understanding of the effectiveness and efficiency of telemedicine in the Saudi Arabian health care system. Finally, this study provides important insights into the perceptions and experiences of health care professionals regarding telemedicine during the COVID-19 pandemic in Saudi Arabia. However, further research is needed to build on these findings and to provide a more comprehensive understanding of the challenges and opportunities associated with telemedicine in the country.

Future research could also include health care professionals who have little or no experience with telemedicine to determine the barriers and challenges that need to be addressed to increase adoption. In addition, further research on the cybersecurity challenges of working from home during the COVID-19 pandemic, as discussed by Sebastian in a descriptive study [37], and persuasive strategies to improve artificial intelligence adoption in health care, as proposed by Sebastian et al [38], would provide valuable insights into addressing potential concerns and optimizing telemedicine systems.

### Conclusions

This study offers an in-depth understanding of health care practitioners’ perceptions, awareness, and experiences related to telemedicine in Saudi Arabia during the COVID-19 pandemic. There was a significant awareness among participants, reflecting the readiness of the health care sector to adapt to technological advances in medical care delivery. Notably, consultants and technicians showed heightened knowledge, underscoring the disparities in exposure and education across professional roles. Echoing global sentiments, our participants affirmed the myriad benefits telemedicine brings to the table. They highlighted its pivotal role in conserving clinician time, streamlining health care provisions, and enhancing the overall quality of patient care. However, the journey of telemedicine’s full-fledged adoption is not without its challenges. Patient privacy and confidentiality emerged as pronounced concerns, especially when contextualized within Saudi Arabia’s unique cultural and regulatory landscape.

The qualitative interviews deepened our understanding by surfacing 5 dominant themes: culture, barriers and challenges, communication strategies, the nuances of implementation, and the essence of continuous evaluation. These themes underline the facets integral to telemedicine’s success. For the technology to fully realize its potential, it is imperative to cultivate an ecosystem characterized by acceptance, adaptability, robust communication, unwavering support, and iterative evaluation.

In the wake of the pandemic, telemedicine has unequivocally demonstrated its value as a cost-effective, quality-enhancing tool that maintains continuity in medical care delivery. Looking ahead, it is essential that stakeholders continually assess and refine telemedicine frameworks, ensuring that they align with evolving health care needs and deliver the highest standards of care.

## Appendix

Multimedia Appendix 1 Survey.

## Data Availability

The data sets generated and analyzed during this study are available from the corresponding author upon reasonable request.
