# Supplementary material for: The Perception of Health Care Practitioners Regarding Telemedicine During COVID-19 in Saudi Arabia: Mixed Methods Study
Source: JMIR Form Res. 2023 Sep 28;7:e47065. doi: 10.2196/47065 (PMC10540916; doi:10.2196/47065)
Supplement: Multimedia Appendix 1 [file formative_v7i1e47065_app1.pdf]

# The perception of healthcare practitioners regarding telemedicine during COVID-19 in Saudi Arabia

| No | Section 1. Demographic Information of the healthcare professionals |
|---|---|
| 1 | **Gender** |
| | ❑ Male |
| | ❑ Female |
| 2 | **Age** |
| | ❑18-30 |
| | ❑31-40 |
| | ❑41-50 |
| | ❑51-60 |
| | ❑60+ |
| 3 | **Nationality** |
| | ❑ Saudi |
| | ❑ Non-Saudi:<br>❑India ❑Pakistan. ❑Egypt. ❑Sudan. ❑Philippine's.<br>❑Syria. ❑Jordan. ❑Sri Lanka. ❑Bangladesh. ❑Others |
| 4 | **Name of the hospital where your employed** |
| | ❑ King Abdul-Aziz university hospital |
| | ❑ King Saud university hospital |
| | ❑ Imam Abdulrahman bin Faisal university hospital |
| 5 | **Which department are you currently working at?** |
| | |
| 6 | **What is your current professional level?** |
| | ❑ Intern |
| | ❑ Resident |
| | ❑ Technician |
| | ❑ Specialist |
| | ❑ Consultant |
| 7 | **Year of experience at your workplace** |
| | ❑ 1 to 2 years |
| | ❑ 3 to 4 years |
| | ❑ 4 to 5 years |
| | ❑ >5 years |
| 8 | **Approximately how many patients have you treated/seen through telemedicine channels per day during Covid-19?** |
| | ❑ Between 20 to 40 patients |
| | ❑ Between 40 to 60 patients |
| | ❑ Between 60 to 80 patients |

| | |
|---|---|
| | ❑ Between 80 to 100 patients |
| | ❑>100 patients |
| 9 | **How many hours do you dedicate for telemedicine service per day during Covid-19?** |
| | ❑ Between 1hr to 3 hrs. |
| | ❑ Between 3hr to 5 hrs. |
| | ❑ Between 5hr to 8 hrs. |
| | ❑ >8 hrs. |
| 10 | **Component of telemedicine used during Covid -19 (Click more than one choice)** |
| | ❑ Consultations |
| | ❑ Monitoring patients. |
| | ❑ Diagnosis |
| | ❑ Treatment |
| | ❑ Nursing |
| 11 | **Mode of telemedicine interaction used during Covid-19 (Click more than one choice)** |
| | ❑ Telephone |
| | ❑ Videoconferencing (ex.: Zoom, Teams, WebEx…etc.) |
| | ❑ Online chatting (Ex. WhatsApp, texting) |
| | ❑ MOH applications (ex. Seha) |
| | ❑ E-mail |
| | ❑ other: |

**Section 2.** Healthcare professional knowledge of telemedicine

| No | Knowledge of Telemedicine | Strongly Agree | Agree | Neutral | Disagree | Strongly Disagree |
|---|---|---|---|---|---|---|
| 1 | I am familiar with telemedicine technology? | ❑ | ❑ | ❑ | ❑ | ❑ |
| 2 | I am familiar with telemedicine guidelines | ❑ | ❑ | ❑ | ❑ | ❑ |
| 3 | continuous training is essential in the use of telemedicine for doctors. | ❑ | ❑ | ❑ | ❑ | ❑ |

**Section 3.** Healthcare professional perception of advantages of using telemedicine technology during Covid-19

| No | Healthcare professional perception of the advantages of telemedicine technology | Strongly Agree | Agree | Neutral | Disagree | Strongly Disagree |
|---|---|---|---|---|---|---|

| | | | | | | |
|---|---|---|---|---|---|---|
| 1 | I am familiar with the benefits of telemedicine. | ❑ | ❑ | ❑ | ❑ | ❑ |
| 2 | Telemedicine is effective in reducing the costs of patient care in hospitals. | ❑ | ❑ | ❑ | ❑ | ❑ |
| 3 | Telemedicine influences users' satisfaction. | ❑ | ❑ | ❑ | ❑ | ❑ |
| 4 | Telemedicine technology save clinicians' time. | ❑ | ❑ | ❑ | ❑ | ❑ |
| 5 | Telemedicine provides faster and better medical care. | ❑ | ❑ | ❑ | ❑ | ❑ |
| 6 | Telemedicine is an effective technology in improving patient care. | ❑ | ❑ | ❑ | ❑ | ❑ |
| **Section 4.** Healthcare professional perception of disadvantages of using telemedicine technology during Covid-19 | | | | | | |
| **No** | **Healthcare professional perception of the disadvantages of telemedicine technology** | **Strongly Agree** | **Agree** | **Neutral** | **Disagree** | **Strongly Disagree** |
| 1 | Telemedicine technology disrupts the doctor–patient relationship. | ❑ | ❑ | ❑ | ❑ | ❑ |
| 2 | Telemedicine technology reduces the effectiveness of patient care. | ❑ | ❑ | ❑ | ❑ | ❑ |
| 3 | Telemedicine technology causes psychological harm to the patients. | ❑ | ❑ | ❑ | ❑ | ❑ |
| 4 | Telemedicine technology results in unauthorized access to patient medical information. | ❑ | ❑ | ❑ | ❑ | ❑ |
| 5 | Telemedicine technology increases hospital expenses. | ❑ | ❑ | ❑ | ❑ | ❑ |
| 6 | Telemedicine technology increases malpractice in healthcare. | ❑ | ❑ | ❑ | ❑ | ❑ |
| **Section 5.** Healthcare professional perception of necessity of using telemedicine technology during Covid-19 | | | | | | |
| **No** | **Healthcare professional perception of the necessity of telemedicine technology** | **Strongly Agree** | **Agree** | **Neutral** | **Disagree** | **Strongly Disagree** |
| 1 | Telemedicine technology is necessary for patient care. | ❑ | ❑ | ❑ | ❑ | ❑ |
| 2 | Telemedicine provides healthcare to patients in a timely manner. | ❑ | ❑ | ❑ | ❑ | ❑ |
| 3 | Telemedicine is essential to provide healthcare to underprivileged and remote areas. | ❑ | ❑ | ❑ | ❑ | ❑ |

| | | | | | | |
|---|---|---|---|---|---|---|
| 4 | Telemedicine technology provides doctors with instant access to patient information. | ❑ | ❑ | ❑ | ❑ | ❑ |
| 5 | National standards are essential for telemedicine technology implementation in Saudi Arabia. | ❑ | ❑ | ❑ | ❑ | ❑ |
| **Section 6.** Healthcare professional perception of issues affecting telemedicine technology during Covid-19 | | | | | | |
| **No** | **Healthcare professional perception of issues affecting telemedicine** | **Strongly Agree** | **Agree** | **Neutral** | **Disagree** | **Strongly Disagree** |
| 1 | Concerns about patient privacy/confidentiality. | ❑ | ❑ | ❑ | ❑ | ❑ |
| 2 | High cost of equipment. | ❑ | ❑ | ❑ | ❑ | ❑ |
| 3 | Negative attitudes of staff involved. | ❑ | ❑ | ❑ | ❑ | ❑ |
| 4 | Lack of user-friendly software | ❑ | ❑ | ❑ | ❑ | ❑ |
| 5 | Lack of suitable training in the use of equipment. | ❑ | ❑ | ❑ | ❑ | ❑ |
| 6 | Perceived increase in workload. | ❑ | ❑ | ❑ | ❑ | ❑ |
| 7 | Lack of perceived clinical usefulness. | ❑ | ❑ | ❑ | ❑ | ❑ |
| **Section 7.** Healthcare professional perception of the effectiveness of telemedicine used by patient during Covid-19 | | | | | | |
| **No** | **Healthcare professional perception perceptions of the effectiveness of telemedicine used by patient** | **Strongly Agree** | **Agree** | **Neutral** | **Disagree** | **Strongly Disagree** |
| 1 | Patients easily use telemedicine tools during consultations. | ❑ | ❑ | ❑ | ❑ | ❑ |
| 2 | Understanding medical problems of patients are clear during consultation. | ❑ | ❑ | ❑ | ❑ | ❑ |
| 3 | Provides appropriate management plans to patients. | ❑ | ❑ | ❑ | ❑ | ❑ |
| 4 | Patients promptly turn up for recall check up | ❑ | ❑ | ❑ | ❑ | ❑ |
